# Parental early life environments drive transgenerational plasticity of offspring metabolism in a freshwater fish (*Danio rerio*)

**DOI:** 10.1098/rsbl.2023.0266

**Published:** 2023-10-04

**Authors:** Melanie D. Massey, Anne C. Dalziel

**Affiliations:** ^1^ Department of Biology Life Science Centre, Dalhousie University, 1355 Oxford Street, PO Box 15000, Halifax, Nova Scotia, Canada B3H 4R2; ^2^ Biology Department, Saint Mary's University, 923 Robie Street, Halifax, Nova Scotia, Canada B3H 3C3

**Keywords:** early life, metabolism, parental effects, plasticity, transgenerational plasticity, zebrafish

## Abstract

Parental experiences can lead to changes in offspring phenotypes through transgenerational plasticity (TGP). TGP is expected to play a role in improving the responses of offspring to changes in climate, but little is known about how the early lives of parents influence offspring TGP. Here, we use a model organism, zebrafish (*Danio rerio*), to contrast the effects of early and later life parental thermal environments on offspring routine metabolism. To accomplish this, we exposed both parents to either constant optimal (27°C) or environmentally realistic diel fluctuating (22–32°C) temperatures during early (embryonic and larval) and later (juvenile and adult) life in a factorial design. We found significant reduction of routine metabolic rates (greater than 20%) at stressful temperatures (22°C and 32°C) after biparental early life exposure to fluctuating temperatures, but little effect of later life parental temperatures on offspring metabolism. This reduction reflects metabolic compensation and is expected to enhance offspring body sizes under stressful temperatures. These changes occur over and above the effects of parental environments on egg size, suggesting alternate non-genetic mechanisms influenced offspring metabolic rates.

## 1. Background

The experiences of parents can impact their offspring through a suite of mechanisms that transcend genetic inheritance, including parental care, offspring provisioning and epigenetic facilitation [1,2]. These non-genetic parental effects, collectively termed ‘transgenerational plasticity’ (TGP), have drawn considerable attention for their role in shaping offspring phenotypic variation and fitness [3,4] and potential to facilitate rapid, beneficial responses to changing environments [2,5,6].

Temperature is a key driver of phenotypic plasticity in ectotherms [7], and continued increases in global thermal means and variance are expected to negatively impact many aspects of organismal performance, including body size via increased metabolic demands as temperatures rise [8–10]. Yet, through TGP, parental exposure to warm temperatures can improve outcomes for offspring experiencing warm temperatures themselves, relative to offspring whose parents received no such cue (‘anticipatory parental effects' [11]; reviewed in [2]). For example, lifelong or reproductive parental acclimation to warm, constant temperatures increases the relative growth rates and body sizes of warm-incubated offspring in fishes [12–17] and is associated with epigenetic increases in offspring metabolic efficiency [12,18]. However, no studies to date have examined TGP in response to variable temperatures, which better reflect natural or predicted thermal conditions. Thermal variability and periodic heating events are expected to intensify with climate change [19,20], and constant versus variable temperatures often produce different phenotypic effects, even when sharing thermal means [21,22]. There is therefore an urgent need to understand how TGP may manifest under ecologically realistic thermal variability [2,23].

The occurrence and strength of TGP can also be influenced by the ontogenetic timing during which parents experience thermal stressors, and most studies have investigated parental exposures during sexual maturity and reproduction [2,24]. However, embryos and larvae may be especially environmentally sensitive, and cellular/molecular changes during early development can cascade into large effects on parental and subsequent offspring phenotypes [6,25,26]. Moreover, beneficial TGP is expected to occur when cues experienced by parents supply accurate information about offspring environments; the windows of parental sensitivity should thus vary depending on the ecology of focal organisms [1,3,11]. In some cases, such as when juvenile and adult habitat use differs, parental early life environments may be better predictors of offspring environments [27,28].

Few studies have compared the transgenerational effects of pre- and post-maturity parental thermal experiences ([2,24]; but see: [29]). In fishes, pre-maturity parental exposure to warm, constant temperatures appears to have beneficial effects on offspring in warm conditions, enhancing body size [30] and aerobic scope [31,32]. Although these studies support the existence of early ontogentic critical windows for TGP, they do not delineate the influence of the earliest parental life stages from later juvenile development. The sensitivity of parental development between fertilization and the juvenile stage is thus of particular interest, especially as these stages are highly responsive to environmentally induced epigenetic modifications [2,25].

Here, we use zebrafish (*Danio rerio*) as a model to investigate how the timing of biparental thermal exposure influences offspring metabolism, by comparing the effects of constant versus challenging diel variable thermal treatments in a 1-year long factorial experiment. We delineate parental ‘early life’ as pre-metamorphic embryonic and larval stages (day 0 to 29), in contrast to ‘later’ post-metamorphic juvenile and adult stages (day 30+). We chose this delineation because pre-metamorphosis is a candidate window for epigenetic effects [25], defined by significant tissue differentiation in teleost fishes [33]. Moreover, the earliest life stages of wild zebrafish occur during the distinctly thermally variable monsoon season (approx. 12–31°C) [34,35]. Zebrafish parental early life environments may therefore act as better cues for young offspring than later life environments (i.e. given environmental autocorrelation between monsoon seasons) [1,28]. We reared parents in thermal treatments for 1 year, simulating their annual life cycle [34], and tested for TGP in routine metabolic rate (RMR) of 1-day old offspring at stressful temperatures (22°C and 32°C). We measured this trait because plasticity-induced reductions in RMR can improve subsequent juvenile growth in stressful thermal conditions, [12,18,36], and juvenile size is a fitness-related trait in teleost fishes [37].

## 2. Methods

### (a) Fish husbandry and thermal treatments

We collected parental generation (F_1_) zebrafish embryos from three non-sibling matings (families) of wild-type-AB fish within 3 h post-fertilization (hpf) from the Dalhousie Zebrafish Core. Grandparental source fish (F_0_) were kept in control standard zebrafish laboratory conditions at 27°C (electronic supplementary material).

**Figure 1.**
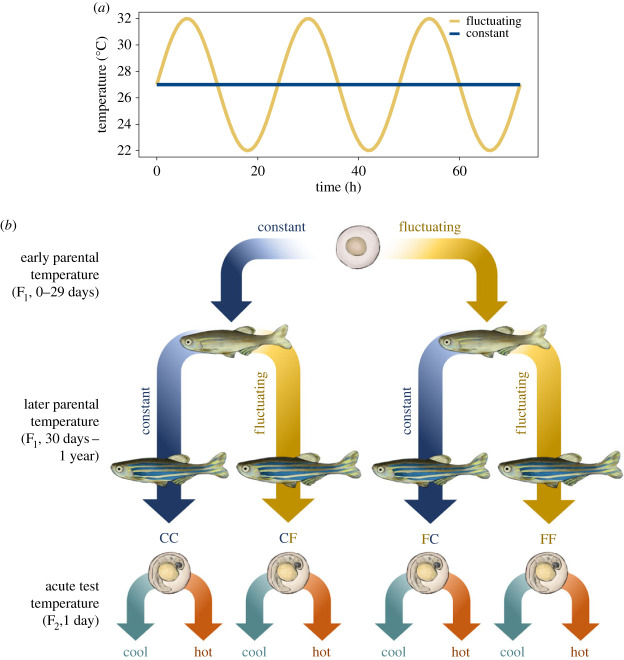
Split-clutch, factorial design of a long-term transgenerational plasticity experiment. (*a*) Constant (‘C’, blue; 27°C) and diel Fluctuating (‘F’, gold; 22–32°C) thermal treatments for F_1_ fish. (*b*) F_1_ zebrafish (*Danio rerio*) were exposed to thermal treatments during Early development (0–29 days; first letter) and Later development (30 days to 1 year; second letter). Offspring (F_2_) routine metabolic rates were then tested at Cool (teal; 22°C) or Hot (red; 32°C) temperatures.

F_1_ embryos were brought to the Dalhousie University Aquatron and each family was divided into two thermal treatment groups (figure 1): a Constant 27°C, which reflects a thermal optimum for reproduction and growth in zebrafish [34,35], and a Fluctuating temperature that varied sinusoidally on a diel basis (22–32°C; figure 1). This regime was intended to reflect natural thermal variability [34], while exposing fish to transient stressful temperatures, minimizing constant temperature-induced pathologies [21,34,38–40].

F_1_ embryos and larvae were reared in their initial ‘Early Parental Temperature’ treatment (Constant or Fluctuating) for 29 dpf (days post-fertilization). At the onset of the juvenile stage (30 dpf), fish were equally split into Constant or Fluctuating temperatures in groups of 12–15, and held in these conditions through sexual maturity (90 dpf) until 1 year of age, establishing a ‘Later Parental Temperature’ treatment (figure 1). In total, there were four groups representing factorial combinations of Early and Later Parental Temperature treatments, with six replicate tanks per group, each with two replicates per family. At 1 year of age, sibling F_1_ fish were bred within each treatment to produce F_2_ embryos, which were kept at 27°C for 24 h (figure 1; electronic supplementary material).

### (b) Embryo metabolism

We measured RMRs of 24 hpf F_2_ embryos at two acute Test Temperatures (22 and 32°C) representing the thermal extremes at which zebrafish develop normally [35]. We used a Microplate Respirometry System (Loligo Systems) with two 24-well, 80 µl microplates run in parallel. Microplates were calibrated to manufacturer specifications the previous night. We photographed individual embryos under a dissecting scope and placed embryos into wells randomly assigned by MicroResp software (Loligo Systems), with four blanks (electronic supplementary material). Microplates were then set to the two Test Temperatures, and the entire system was placed on an orbital shaker on the lowest setting, reducing oxygen stratification. We recorded oxygen consumption in each microplate for at least 3 h. We repeated measurements across six trials, with each trial day consisting of offspring from one of the three F_1_ families, and each of the four parental thermal treatment combinations.

### (c) Statistical analyses

We used MicroResp software to estimate embryo RMR in normoxia (80–100% oxygen saturation). We selected an *r*^2^ value of ≥0.8 for linear estimations of oxygen consumption (MO_2_) rates, and applied automatic correction for background respiration from blanks.

We measured F_1_ embryo egg diameter from photographs as a proxy for offspring size. We could not consistently measure yolk diameters, as yolks were occasionally obscured by embryos; however, we previously showed a strong positive correlation between yolk and egg diameters across thermal treatments [41]. Upon reviewing images, we excluded embryos that exhibited deformity (e.g. kyphosis) or had visible tears in their chorions.

We used Bayesian generalized linear mixed models to analyse data using the *brms* package (v. 2.19.0 [42]) in R (v. 4.2.2). These models estimated the causal effect of predictors on offspring RMR. For Model 1, we estimated the effects of ‘Early’ (0–29 dpf) and ‘Later’ (30 + dpf) Parental Temperatures (Constant or Fluctuating) as well as the acute ‘Test’ Temperature (22 or 32°C) on log-transformed embryo RMR [43]. We included interactions between ‘Test’ Temperature and each Parental Temperature treatment, to test for differences in TGP thermal sensitivity. We included ‘Trial’ as a random intercept to account for within-trial interdependence (e.g. due to the sum of differences in daily calibration and family level effects). To test whether changes in offspring RMR were mediated via changes to egg size, we also fitted Model 2, correcting for ‘Egg Size’ (diameter). Using two separate models allowed us to avoid overcontrol bias [40] in our estimation of ‘Later’ Parental Temperature in Model 1 as this treatment impacts egg size [41]. We interpreted non-negligible effects of Early or Later Parental Temperatures on offspring RMR as evidence of TGP, and interactions between Parental Temperatures and Test Temperatures as differences in the thermal sensitivity of TGP between Test Temperatures. Model specifications are detailed in electronic supplementary material.

## 3. Results

**Figure 2.**
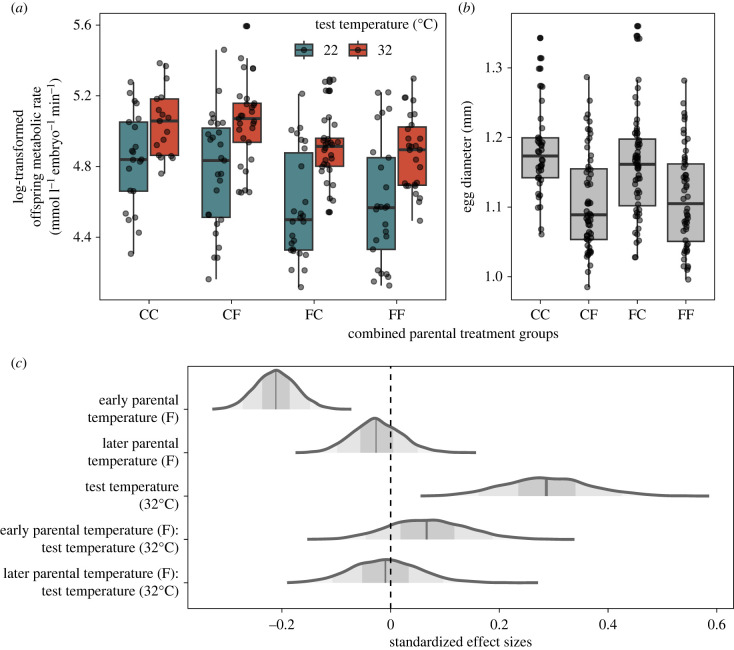
Transgenerational plasticity in offspring metabolism of F_1_ zebrafish (*Danio rerio*) exposed to Constant (‘C’, 27°C) or diel Fluctuating (‘F’, 22–32°C) temperatures during Early Parental development (0–29 days, first letter), Later Parental development (30 + days, second letter), or both. (*a*) F_2_ offspring routine metabolic rates (RMR) tested at Cool (teal; 22°C) or Hot (red; 32°C) thermal extremes. (*b*) Offspring egg diameter. (*c*) Posterior distributions of effect sizes of Parental Temperatures, Test Temperature and their interactions on F_2_ offspring RMR. Posterior means are represented by thin dark lines at the mean of each distribution; 90% and 50% uncertainty intervals are represented by increasingly light shading.

Median results for metabolism and egg size, with individual data circled, are illustrated in figure 2*a,b*, and posterior effect sizes for Model 1 are illustrated in figure 2*c*. Briefly, mean effect sizes further from 0 indicate stronger effects of predictors on offspring RMR, and the greater the overlap of uncertainty intervals (UIs) with 0, the less certainty in estimates.

As expected, we found large differences in F_2_ offspring RMR at the two acute Test Temperatures, with 28.4% (90% UIs: [13.93%, 44.8%]) higher metabolic rates at 32°C compared to 22°C (figure 2*a,c*). Early Parental Fluctuating Temperatures led to a decrease in embryo RMR, with 21.3% lower RMR when compared to offspring from Early Parental Constant Temperatures (figure 2*a,c*; 90% UIs: [−28.3%, −14.0%]). In comparison, Later Parental Temperature had a weak effect on offspring RMR, with 2.7% lower RMR in offspring of Fluctuating versus Constant temperatures (figure 2*a,c*; 90% UIs: [−11.1%, 6.5%]).

We also detected a weak interaction between Early Parental Temperature and the acute Test Temperature, such that the offspring of parents exposed to Early Fluctuating Temperatures were 7.0% less sensitive (90% UIs: [−5.8%, 21.7%]) at the acute 32°C Test Temperature, when compared to 22°C (figure 2*a,c*). We detected no significant interaction between Later Parental Temperature and Test Temperature (figure 2*a,c*; median: 1.0%, 90% UIs: [−13.2%, 12.7%]).

There was a strong influence of egg diameter (figure 2*b*) on RMR, such that a 1 mm increase in diameter was predicted to lead to a 70% increase in RMR, with wide variation in the posterior distribution (Model 2; 90% UIs: [4.7%, 178.3%]; details in electronic supplementary material, Results). However, the inclusion of this predictor did not meaningfully impact estimates of Early Parental Temperature's effects, although the lowering effect of Later Parental Fluctuating Temperature on offspring RMR disappeared (median: 1.4%, 90% UIs:[−8.0%, 11.3%]).

## 4. Discussion

Although TGP is an important mechanism through which organisms can modify offspring phenotypes to withstand climatic stressors, we have few empirical data describing the role of parents' early life thermal experiences on offspring responses [24]. Here, we show that biparental thermal experiences of F_1_ zebrafish during their first month of life strongly affected F_2_ offspring embryonic routine metabolic rates when exposed to stressful temperatures. Indeed, metabolic rates were over 20% lower at thermal extremes when parents were initially reared in challenging, thermally variable conditions compared to optimal, constant early life conditions.

Anthropogenic climate warming has increased ectotherm metabolic rates worldwide [10,36], leading to increases in energetic demands and ultimately imposing constraints on adult body sizes [8,9]. Although interpretations of plasticity-induced reductions in metabolism are context dependent [44], lower metabolic rates are predicted to confer growth benefits under stressful thermal environments [18,36]. Although later offspring body sizes were not measured here, other studies rearing fish in warm temperatures have explicitly linked lower routine metabolic rates to improved growth rates via TGP [12,18] and within-generation plasticity [45]. Therefore, we interpret the reduction in embryonic metabolic rate as beneficial metabolic compensation, which could ameliorate negative impacts of thermal stressors on body size as offspring develop [12,18,44,45].

We also tested whether these reductions in metabolic rate were mediated via direct reductions in egg size, given that metabolic rates positively scale with organismal size [46] and later life fluctuating temperatures reduce both parental body sizes and egg provisioning in zebrafish [41]. By correcting for differences in egg size, the small decrease in offspring RMR owing to fluctuating later life parental temperatures disappeared. This may reflect a condition-transfer effect [47], in which constraints on parental energy expenditure during later life led to a reduction in egg size, and thus offspring RMR [11,41]. By contrast, the large reduction in RMR caused by fluctuating early parental temperatures occurred over and above effects on egg size. Instead, it is likely that this metabolic compensation was facilitated through alternative epigenetic mechanisms established during parental early life [25]. For example, beneficial TGP to warm temperatures has been shown to be mediated through differential gene expression, including the upregulation of genes associated with mitochondrial activity and energy production [15,32,48]. Other potential mechanisms include methylation and histone modifications in germline cells during early parental development [6,24,25].

In this experiment, we also found a modest interaction between early parental temperatures and acute test temperatures. Contrary to previous findings, however, we found slightly less metabolic reduction at the hotter versus the cooler offspring test temperature for offspring from early fluctuating temperature parents [12,30]. It is possible that the scope for thermal sensitivity of TGP was reduced in the acute hot test temperature because of physiological limitations, as 32°C is the upper thermal limit for the normal development of zebrafish embryos [35].

Overall, we found that there is strong TGP in offspring routine metabolic rate in response to ecologically realistic thermal variability experienced during parental early life, but a negligible influence of later life adult thermal experiences. These data highlight the importance of studying how the timing of stressors influences plasticity, and show that early life parental environments can generate significant phenotypic variation in subsequent generations. A goal of future studies will be to understand the long-term consequences of these changes.

## Data Availability

Data and code for this piece are available from the Dryad Digital Repository: https://doi.org/10.5061/dryad.sqv9s4n87 [49]. The data are provided in the electronic supplementary material [50].

## References

[1] Uller T. 2008 Developmental plasticity and the evolution of parental effects. Trends Ecol. Evol. **23**, 432-438. (10.1016/j.tree.2008.04.005) 18586350

[2] Donelson JM, Salinas S, Munday PL, Shama LNS. 2018 Transgenerational plasticity and climate change experiments: where do we go from here? Glob. Change Biol. **24**, 13-34. (10.1111/gcb.13903) 29024256

[3] Uller T, Nakagawa S, English S. 2013 Weak evidence for anticipatory parental effects in plants and animals. J. Evol. Biol. **26**, 2161-2170. (10.1111/jeb.12212) 23937440

[4] Mousseau TA, Fox CW. 1998 Maternal effects as adaptations. Oxford, UK: Oxford University Press.

[5] Salinas S, Brown SC, Mangel M, Munch SB. 2013 Non-genetic inheritance and changing environments. Non-Genetic Inherit. **1**, 38-50. (10.2478/ngi-2013-0005)

[6] Donelan SC, Hellmann JK, Bell AM, Luttbeg B, Orrock JL, Sheriff MJ, Sih A. 2020 Transgenerational plasticity in human-altered environments. Trends Ecol. Evol. **35**, 115-124. (10.1016/j.tree.2019.09.003) 31706627 PMC9440440

[7] Angilletta MJ. 2009 Thermal adaptation: a theoretical and empirical synthesis. New York, NY: Oxford University Press.

[8] Riemer K, Anderson-Teixeira KJ, Smith FA, Harris DJ, Ernest SKM. 2018 Body size shifts influence effects of increasing temperatures on ectotherm metabolism. Global Ecol. Biogeogr. **27**, 958-967. (10.1111/geb.12757)

[9] Schulte PM. 2015 The effects of temperature on aerobic metabolism: towards a mechanistic understanding of the responses of ectotherms to a changing environment. J. Exp. Biol. **218**, 1856-1866. (10.1242/jeb.118851) 26085663

[10] Dillon ME, Wang G, Huey RB. 2010 Global metabolic impacts of recent climate warming. Nature **467**, 704-706. (10.1038/nature09407) 20930843

[11] Marshall DJ, Uller T. 2007 When is a maternal effect adaptive? Oikos **116**, 1957-1963. (10.1111/j.2007.0030-1299.16203.x)

[12] Shama LNS, Strobel A, Mark FC, Wegner KM. 2014 Transgenerational plasticity in marine sticklebacks: maternal effects mediate impacts of a warming ocean. Funct. Ecol. **28**, 1482-1493. (10.1111/1365-2435.12280)

[13] Salinas S, Munch SB. 2012 Thermal legacies: transgenerational effects of temperature on growth in a vertebrate. Ecol. Lett. **15**, 159-163. (10.1111/j.1461-0248.2011.01721.x) 22188553

[14] Donelson JM, Munday PL, McCormick MI. 2012 Climate change may affect fish through an interaction of parental and juvenile environments. Coral Reefs **31**, 753-762. (10.1007/s00338-012-0899-7)

[15] Shama LNS, Mark FC, Strobel A, Lokmer A, John U, Mathias Wegner K. 2016 Transgenerational effects persist down the maternal line in marine sticklebacks: gene expression matches physiology in a warming ocean. Evol. Appl. **9**, 1096-1111. (10.1111/eva.12370) 27695518 PMC5039323

[16] Shama LNS, Wegner KM. 2014 Grandparental effects in marine sticklebacks: transgenerational plasticity across multiple generations. J. Evol. Biol. **27**, 2297-2307. (10.1111/jeb.12490) 25264208

[17] Donelson JM, Wong M, Booth DJ, Munday PL. 2016 Transgenerational plasticity of reproduction depends on rate of warming across generations. Evol Appl **9**, 1072-1081. (10.1111/eva.12386) 27695516 PMC5039321

[18] Donelson JM, Munday PL, McCormick MI, Pitcher CR. 2012 Rapid transgenerational acclimation of a tropical reef fish to climate change. Nat. Clim. Change **2**, 30-32. (10.1038/nclimate1323)

[19] Bathiany S, Dakos V, Scheffer M, Lenton TM. 2018 Climate models predict increasing temperature variability in poor countries. Sci. Adv. **4**, eaar5809. (10.1126/sciadv.aar5809) 29732409 PMC5931768

[20] Meehl GA, Tebaldi C. 2004 More intense, more frequent, and longer lasting heat waves in the 21st century. Science **305**, 994-997. (10.1126/science.1098704) 15310900

[21] Massey MD, Hutchings JA. 2021 Thermal variability during ectotherm egg incubation: a synthesis and framework. J. Exp. Zool. **335**, 59-71. (10.1002/jez.2400) 32767534

[22] Huey R, Berrigan D. 1996 Testing evolutionary hypotheses of acclimation. In Animals and temperature: phenotypic and evolutionary adaptation, pp. 203-237. Cambridge, UK: Society of Experimental Biology.

[23] Vasseur DA, DeLong JP, Gilbert B, Greig HS, Harley CDG, McCann KS, Savage V, Tunney TD, O'Connor MI. 2014 Increased temperature variation poses a greater risk to species than climate warming. Proc. R. Soc. B **281**, 1-8. (10.1098/rspb.2013.2612) PMC3924069 24478296

[24] Burton T, Metcalfe NB. 2014 Can environmental conditions experienced in early life influence future generations? Proc. R. Soc. B **281**, 20140311. (10.1098/rspb.2014.0311) PMC4024293 24807254

[25] Nagy C, Turecki G. 2012 Sensitive periods in epigenetics: bringing us closer to complex behavioral phenotypes. Epigenomics. **4**, 445-457. (10.2217/epi.12.37) 22920183 PMC5293543

[26] Beldade P, Mateus ARA, Keller RA. 2011 Evolution and molecular mechanisms of adaptive developmental plasticity. Mol. Ecol. **20**, 1347-1363. (10.1111/j.1365-294X.2011.05016.x) 21342300

[27] Taborsky B. 2006 The influence of juvenile and adult environments on life-history trajectories. Proc. R. Soc. B. **273**, 741-750. (10.1098/rspb.2005.3347) PMC1560075 16608695

[28] Taborsky B. 2006 Mothers determine offspring size in response to own juvenile growth conditions. Biol. Lett. **2**, 225-228. (10.1098/rsbl.2005.0422) 17148368 PMC1618922

[29] Fischer K, Eenhoorn E, Bot ANM, Brakefield PM, Zwaan BJ. 2003 Cooler butterflies lay larger eggs: developmental plasticity versus acclimation. Proc. R. Soc. Lond. B **270**, 2051-2056. (10.1098/rspb.2003.2470) PMC1691478 14561294

[30] Shama LNS. 2015 Bet hedging in a warming ocean: predictability of maternal environment shapes offspring size variation in marine sticklebacks. Glob. Change Biol. **21**, 4387-4400. (10.1111/gcb.13041) 26183221

[31] Bernal MA, Ravasi T, Rodgers GG, Munday PL, Donelson JM. 2022 Plasticity to ocean warming is influenced by transgenerational, reproductive, and developmental exposure in a coral reef fish. Evol. Appl. **15**, 249-261. (10.1111/eva.13337) 35233246 PMC8867710

[32] Bernal MA, Donelson JM, Veilleux HD, Ryu T, Munday PL, Ravasi T. 2018 Phenotypic and molecular consequences of stepwise temperature increase across generations in a coral reef fish. Mol. Ecol. **27**, 4516-4528. (10.1111/mec.14884) 30267545

[33] McMenamin SK, Parichy DM. 2013 Metamorphosis in teleosts. Curr. Top. Dev. Biol. **103**, 127-165. (10.1016/B978-0-12-385979-2.00005-8) 23347518 PMC5606158

[34] Spence R, Gerlach G, Lawrence C, Smith C. 2008 The behaviour and ecology of the zebrafish, *Danio rerio*. Biol. Rev. Camb. Philos. Soc. **83**, 13-34. (10.1111/j.1469-185X.2007.00030.x) 18093234

[35] Scott GR, Johnston IA. 2012 Temperature during embryonic development has persistent effects on thermal acclimation capacity in zebrafish. Proc. Natl Acad. Sci. USA **109**, 14 247-14 252. (10.1073/pnas.1205012109) PMC3435178 22891320

[36] Seebacher F, White CR, Franklin CE. 2015 Physiological plasticity increases resilience of ectothermic animals to climate change. Nat. Clim. Change **5**, 61-66. (10.1038/nclimate2457)

[37] Sogard SM. 1997 Size-selective mortality in the juvenile stage of teleost fishes: a review. Bull. Mar. Sci. **60**, 1129-1157.

[38] Morash AJ, Neufeld C, MacCormack TJ, Currie S. 2018 The importance of incorporating natural thermal variation when evaluating physiological performance in wild species. J. Exp. Biol. **221**, jeb164673. (10.1242/jeb.164673) 30037965

[39] Woods HA, Harrison JF. 2002 Interpreting rejections of the beneficial acclimation hypothesis: when is physiological plasticity adaptive? Evolution **56**, 1863-1866. 12389732 10.1111/j.0014-3820.2002.tb00201.x

[40] Arif S, Massey MDB. 2023 Reducing bias in experimental ecology through directed acyclic graphs. Ecol. Evol. **13**, e9947. (10.1002/ece3.9947) 37006894 PMC10050842

[41] Massey MD, Fredericks MK, Malloy D, Arif S, Hutchings JA. 2022 Differential reproductive plasticity under thermal variability in a freshwater fish (*Danio rerio*). Proc. R. Soc. B **289**, 20220751. (10.1098/rspb.2022.0751) PMC9449469 36069011

[42] Bürkner PC et al. 2023 brms: Bayesian regression models using ‘Stan’. See https://CRAN.R-project.org/package=brms.

[43] Gelman A, Hill J. 2007 Data analysis using regression and multilevel/hierarchical models. Cambridge, UK: Cambridge University Press.

[44] Norin T, Metcalfe NB. 2019 Ecological and evolutionary consequences of metabolic rate plasticity in response to environmental change. Phil. Trans. R. Soc. Lond B **374**, 20180180. (10.1098/rstb.2018.0180) 30966964 PMC6365862

[45] Donelson JM, Munday PL, Mccormick MI, Nilsson GE. 2011 Acclimation to predicted ocean warming through developmental plasticity in a tropical reef fish. Glob. Change Biol. **17**, 1712-1719. (10.1111/j.1365-2486.2010.02339.x)

[46] Jerde CL, Kraskura K, Eliason EJ, Csik SR, Stier AC, Taper ML. 2019 Strong evidence for an intraspecific metabolic scaling coefficient near 0.89 in fish. Front. Physiol. **10**, 1166. (10.3389/fphys.2019.01166) 31616308 PMC6763608

[47] Bonduriansky R, Crean AJ. 2018 What are parental condition-transfer effects and how can they be detected? Methods Ecol. Evol. **9**, 450-456. (10.1111/2041-210X.12848)

[48] Veilleux HD et al. 2015 Molecular processes of transgenerational acclimation to a warming ocean. Nat. Clim. Change **5**, 1074-1078. (10.1038/nclimate2724)

[49] Massey MD, Dalziel AC. 2023 Data from: Parental early life environments drive transgenerational plasticity of offspring metabolism in a freshwater fish (*Danio rerio*). *Dryad Digital Repository*. (10.5061/dryad.sqv9s4n87) PMC10547547 37788714

[50] Massey MD, Dalziel AC. 2023 Parental early life environments drive transgenerational plasticity of offspring metabolism in a freshwater fish (*Danio rerio*). Figshare. (10.6084/m9.figshare.c.6858084) PMC10547547 37788714

